# Plasma corin levels provide minimal prognostic utility in left atrial reverse remodeling after catheter ablation of atrial fibrillation: an observational study

**DOI:** 10.3389/fendo.2025.1717422

**Published:** 2025-11-24

**Authors:** Qianqian Wang, Yang Li, Xiaoyang Yuan, Yue Gong, Keyi Song, Chunkai Li, Jinqiu Liu, Yichang Zhao, Feifei Chen

**Affiliations:** 1 Department of Cardiology, The First Affiliated Hospital of Dalian Medical University, Dalian, China; 2 Department of Laboratory Medicine, The First Affiliated Hospital of Dalian Medical University, Dalian, China; 3 Department of Cardiology, Central Hospital of Dalian University of Technology, Dalian, China

**Keywords:** atrial fibrillation, corin, catheter ablation, left atrial reverse remodeling, left atrial volume index

## Abstract

**Objective:**

This study aims to assess the impact of plasma corin levels on left atrial reverse remodeling (LARR) in patients with atrial fibrillation (AF) after catheter ablation (CA).

**Methods:**

This prospective, single-center, observational study included 335 patients undergoing first CA of AF. All patients underwent at least two echocardiographic examinations at intervals of ≥3 months. Corin was measured before and 1 day after CA. LARR was defined as a reduction of ≥20% in left atrial volume index.

**Results:**

After a median of 29.53 months of follow-up, 94 patients exhibited LARR. The plasma corin concentrations drawn pre- and post-procedure in the LARR group were 442.85 [241.25, 631.68] and 516.83 [362.4,697.84] pg/mL, and those were significantly higher than those in the non-LARR group (393.82 [207.09, 558.11] and 473.77 [320.88, 665.75] pg/mL; *P* = 0.036 and *P* = 0.047). The Kaplan–Meier survival curve showed high baseline corin concentrations which indicated a higher rate of LARR than low corin concentrations (*P* = 0.014). The Cox regression found plasma corin levels before or after the procedure were not associated with LARR in AF patients after CA, with or without adjusting for confounding factors. Absence of hypertension (*P* = 0.001, OR = 0.33) and diabetes mellitus (*P* = 0.007, OR = 0.269) was associated with a greater likelihood of LARR in AF patients after CA.

**Conclusions:**

Although plasma corin concentrations were increased in the LARR group, it provided minimal prognostic utility in LARR after CA of AF.

## Introduction

Atrial fibrillation (AF) is a common cardiac arrhythmia that increases mortality and morbidity worldwide (1) and which promotes left atrial (LA) electrical and structural remodeling. However, atrial remodeling has led to atrial cardiomyopathy, setting the stage for AF development. Expert consensus on atrial cardiomyopathies focuses on definition, characterization, and clinical implications (2). Catheter ablation (CA) for AF is an important rhythm-control strategy in symptomatic patients with paroxysmal or persistent AF (PeAF) who are refractory or intolerant to antiarrhythmic drugs; it is, by far, the most common cardiac ablation procedure performed worldwide (3). The restoration of sinus rhythm (SR) contributes to the amelioration of LA structural changes, namely, LA reverse remodeling (LARR), which is an important surrogate marker for AF-free survival (4–6). In addition, LARR could also improve cardiac function and reduce the risk of thromboembolism (5, 7, 8). Therefore, LARR has had an important clinical influence (9). Echocardiography is the most widely used method to evaluate LA dimension and function; LA volume (LAV) is a marker of LA structural remodeling. However, LARR following CA varies substantially among individuals (6, 10).

It has been shown that some biomarkers, such as atrial natriuretic peptide (ANP) (9, 10), B-type natriuretic peptide (BNP), N-terminal pro-BNP (NT-proBNP) (11, 12), C-reactive protein (13), suppression of tumorigenicity 2/interleukin 33-receptor (14), and high-sensitivity cardiac troponin T (15), have been evaluated as predictors of LARR. Corin is a type II transmembrane serine protease highly expressed in cardiomyocytes and converts proANP/proBNP into mature ANP/BNP (16, 17). Recently, a series of studies has demonstrated that corin is associated with cardiovascular diseases, including hypertension, heart failure (HF), acute myocardial infarction, AF, and stroke (18). Soluble corin levels are increased in AF patients (19), and elevated plasma corin levels at baseline have been strongly associated with an increased incidence of AF recurrence after CA (20). However, whether soluble corin levels can predict LARR after CA of AF has not been studied. Therefore, the aim of this study was to investigate the impact of plasma corin levels (measured before and after CA for AF) on LARR after the initial CA of AF.

## Methods

We conducted a single‐center, observational study at the First Affiliated Hospital of Dalian Medical University from April 2019 through May 2023. The study protocol was approved by the hospital’s ethics committee (no. PJ-KS-KY-2025-510) and adhered to the guidelines set forth by the Declaration of Helsinki. All patients signed informed consent forms prior to enrollment.

The study population, clinical measurements, definition of explanatory variables, ablation procedure, medications, and plasma corin measurements were described in a previous study (20). The participants’ peripheral venous blood samples were collected into sodium citrate coagulation test tubes before and 1 day after CA.

### Echocardiographic measurements

Transthoracic echocardiography (TTE) was performed using a commercially available system, EPIQ 7 (Koninklijke Philips N.V.) or Vivid E95 (GE Vingmed Ultrasound). Each patient was analyzed by the same operator blinded to baseline characteristics and clinical outcomes at baseline and follow-up evaluations. TTE measurements were performed according to the recommendations of the American Society of Echocardiography (21, 22). In the case of irregular rhythm, the parameters were measured over 10 beats to avoid bias given by beat-to-beat variability. Left ventricular end‐diastolic diameter (LVEDD) and left ventricular ejection fraction (LVEF) were assessed using the biplane Simpson’s rule. LAV was assessed by the ellipsoid method (23), which requires three LA orthogonal diameters in end-systole just before mitral valve opening. LA diameter (LAD) is the anteroposterior diameter of the LA and was defined in the parasternal long-axis view. LA left-right diameter (LALRD) and LA superior–inferior diameter (LASID) were measured in the apical four-chamber view (24).

The formula for the ellipsoid method is LAV = π/6 × LAD × LALRD × LASID (25, 26). LAV index (LAVI) was calculated as (LAV)/body surface area (BSA), where BSA (m^2^) = 0.007184 × height (cm)^0.725^ × weight (kg)^0.425^. The LA sphericity index (LASI) was calculated as the ratio of LALRD and LASID, where body mass index (BMI) = weight (kg)/height(m)^2^. All patients underwent at least two echocardiographic examinations. When more than two tests were available, the first and last assessments were used to calculate the LARR. The time interval between the two examinations was at least 3 months (5).

### Follow‐up and assessment of LARR

All patients were followed up at regular predefined intervals at our cardiology clinic or with their referring physician, with additional visits as required. Patients who did not attend regular visits were contacted by telephone. AF recurrence was defined as after a 3-month blanking period from the CA following a documented (electrocardiogram, Holter monitoring) episode of AF, atrial tachycardia, or atrial flutter (AFL) lasting >30 s (21). The study population was divided into two groups according to the extent of decrease in the LAVI during a follow-up period calculated from the second echocardiography examination until AF recurrence or until December 31, 2024. The LARR group was defined as patients who exhibited ≥20% decrease in the LAVI, inversely as the non-LARR group (27, 28).

### Statistical analysis

Statistical analysis was performed using the Statistical Package for the Social Sciences (SPSS), version 27.0 (IBM, Armonk, NY, USA), and GraphPad Prism, version 9 (GraphPad Software, San Diego, CA, USA). Categorical variables [sex, PaAF, hypertension, type 2 diabetes mellitus (T2DM), coronary artery disease, HF, left ventricular hypertrophy, and medications] were expressed as percentages and were compared using Pearson’s chi-square (*χ*^2^). Continuous variables (age, BMI, BSA, estimated glomerular filtration rate, lipid factors, BNP, corin, interval time, electrocardiogram parameters, and TTE parameters) were expressed as median [25th percentile (quartile 1)–75th percentile (quartile 3)] and compared using Mann–Whitney test. Receiver operating characteristic (ROC) curves and Youden index were constructed to identify the threshold of corin that best predicted LARR. The patients were categorized based on their corin concentrations according to whether the concentration was above or below the threshold value. The threshold values differed according to sex and AF type. Survival curves were generated using Kaplan–Meier analysis, with a log-rank test assessing the differences.

Cox regression analyses—adjusted for hypertension, AF type, PR interval, corrected QT (QTc) interval, T2DM, HF, and corin levels before or after CA, and TTE parameters, which were statistically different at baseline between the LARR group and non-LARR group—were performed for the primary end point with corin as continuous and dichotomous variables (low *vs*. high concentrations). The continuous variables, including corin values, were normalized by Z-score normalization; one standard deviation was used to calculate the hazard ratio (HR). Binary logistic regression analysis (also adjusted for the variables) was performed for the independent predictors of LARR after CA. The statistical tests were two-sided, and *P* < 0.05 was considered statistically significant.

## Results

### Baseline characteristics

**Figure 1:**
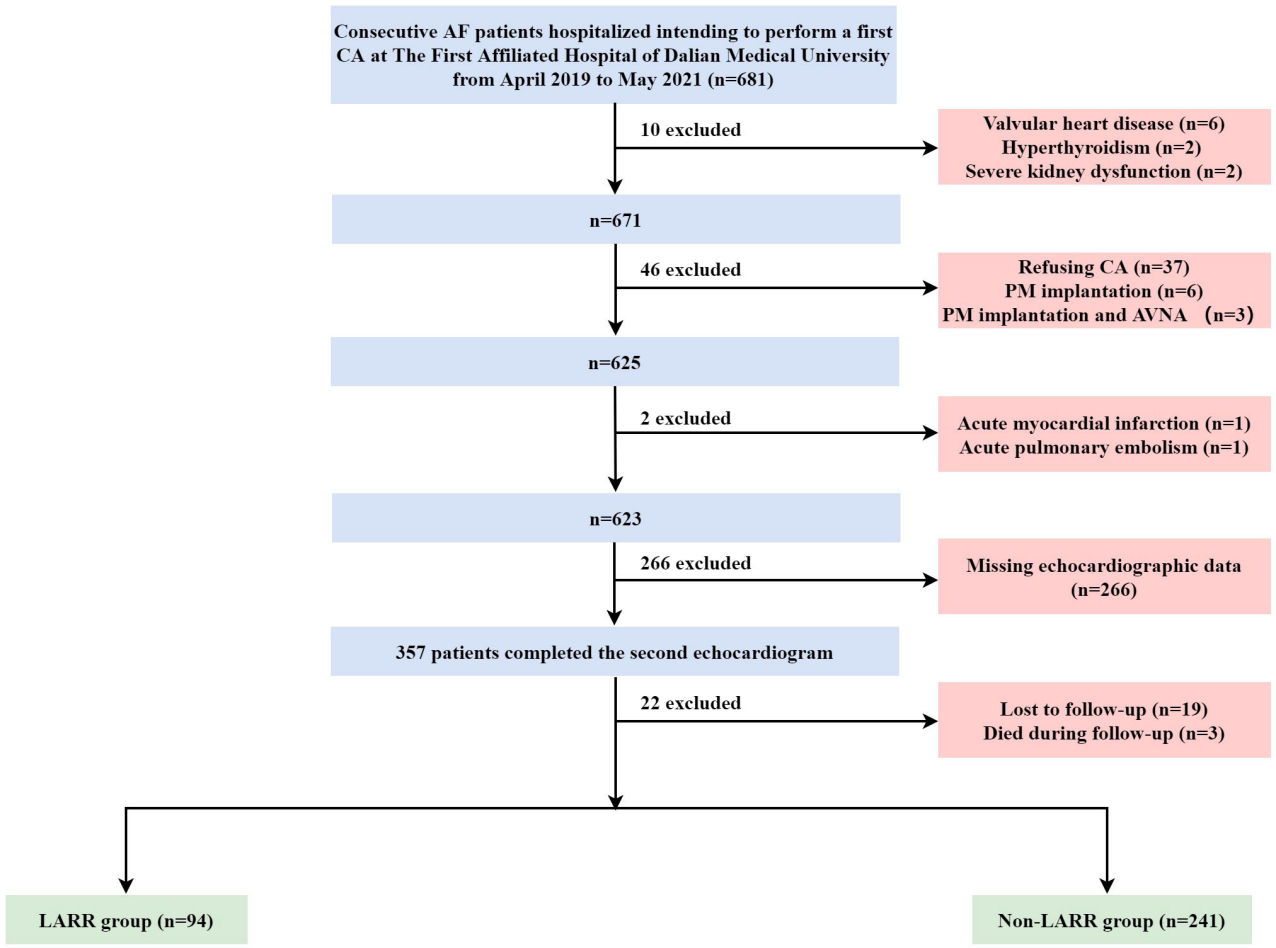
Flow diagram of the inclusion and exclusion of study subjects. AF, atrial fibrillation; CA, catheter ablation; PM, pacemaker; AVNA, atrioventricular nodal ablation; LARR, left atrial reverse remodeling.

This study initially recruited 681 inpatients who intended to undergo their first CA for AF. Of these, 343 cases were excluded because they met the exclusion criteria. Patients with valvular heart disease (*n* = 6), hyperthyroidism (*n* = 2), and severe kidney dysfunction (*n* = 2) were excluded. Patients who refused to undergo the CA procedure (*n* = 37), have undergone pacemaker implantation (*n* = 6), and have undergone concurrent pacemaker implantation and atrioventricular nodal ablation (*n* = 3) were further excluded. Also excluded were one patient diagnosed with acute MI, one patient diagnosed with acute pulmonary embolism, 266 patients with missing echocardiography data, 19 patients who did not complete follow‐up, and three patients who died during follow‐up. Consequently, 335 patients remained, with 94 cases in the LARR group and 241 in the non-LARR group. The flowchart of identification, inclusion, and exclusion is shown in **Figure 1**.

The baseline characteristics of the study population are displayed in **Table 1**. The average age was 65 (57, 69) years; 59.4% were men, and 58.21% had paroxysmal AF (PaAF). During the follow-up period, 105 patients (31.34%) had recurrent AF. Based on a LAVI reduction ≥20%, 94 (28.06%) patients were assigned to the LARR group and 241 (71.94%) to the non-LARR group. Overall, compared with the non-recurrence group, patients in the recurrence group were predominantly male, had higher BSA and plasma corin levels before and after CA, and had a lower frequency of left ventricular hypertrophy. Compared with the non-LARR group, patients in the LARR group had lower frequencies of PaAF, hypertension, and T2DM. In addition, they had a higher frequency of HF, longer PR and QTc intervals, and higher plasma corin levels before and after CA. Moreover, they showed higher LAD, LALRD, LASID, LAV, LAVI, and LVEDD and lower LVEF than the non-LARR group before CA. However, LAD, LALRD, LASID, LAV, and LAVI were reversed in the LARR group after CA. Notably, the interval between the two TTE tests was shorter for the LARR group.

**Table 1:** Baselines and corin concentrations of the total cohort, non-recurrence or recurrence group, non-LARR or LARR group of AF after CA.

Variables	Total cohort (n=335)	Recurrence group (n=105)	Non-recurrence group (n=230)	*P* value	LARR group (n=94)	Non-LARR group (n=241)	*P* value
Age, yrs	65 [57, 69]	63 [57, 66]	65 [57, 70]	0.05	66 [60, 68.5]	66.5 [62.25, 71.75]	0.064
Male, n(%)	199 (59.4)	72 (68.57)	127 (55.22)	0.021	60 (63.83)	139 (57.68)	0.303
BMI, kg/m^2^	25.4 [23.66, 27.72]	25.61 [24.1, 27.73]	25.3 [23.44, 27.69]	0.281	25.69 [23.54, 27.23]	25.71 [24.16, 28.53]	0.457
BSA, m^2^	1.83 [1.71, 1.95]	1.87 [1.75, 2.00]	1.82 [1.7, 1.94]	0.029	1.87 [1.76, 2.01]	1.81 [1.69, 1.93]	0.877
History							
PaAF, n(%)	195 (58.21)	62 (59.03)	133 (57.83)	0.833	38 (40.43)	157 (65.15)	<0.0001
Hypertension, n(%)	177 (52.84)	53 (50.48)	124 (53.91)	0.559	35 (37.23)	142 (58.92)	<0.0001
LVH, n(%)	89 (26.57)	17 (16.19)	72 (31.3)	0.004	18 (19.15)	71 (29.46)	0.055
T2DM, n(%)	73 (21.79)	16 (15.24)	57 (24.78)	0.05	11 (11.7)	62 (25.73)	0.005
HF, n(%)	58 (17.31)	13 (12.38)	45 (19.57)	0.127	25 (26.6)	32 (13.28)	0.004
CAD, n(%)	47 (14.03)	12 (11.43)	35 (15.23)	0.354	11 (11.7)	36 (14.94)	0.444
Laboratory values							
eGFR, ml/min/1.73m^2^	92.01 [82.86, 98.08]	91.00[79.02, 197.87]	90.36 [80.36, 97.41]	0.064	90.45 [80.77, 97.07]	91.12 [81.7, 97.41]	0.764
TC, mmol/L	4.23 [3.5, 4.98]	4.23 [3.3, 5.06]	4.11 [3.31, 4.9]	0.307	4.17 [3.23, 5.23]	3.75 [3.3, 4.82]	0.122
TG, mmol/L	1.23 [0.94, 1.71]	1.23 [1.09, 1.86]	1.2 [0.85, 1.59]	0.149	1.34 [0.87, 1.9]	1.19 [0.98, 1.76]	0.549
HDL-C, mmol/L	1.05 [0.89, 1.24]	0.97 [0.87, 1.28]	1.11 [0.89, 1.26]	0.098	1.06 [0.84, 1.22]	1.07 [0.87, 1.25]	0.902
LDL-C, mmol/L	2.26[1.71,2.78]	2.34[1.62, 2.8]	2.04[1.64, 2.78]	0.258	2.35 [1.55, 2.93]	1.97 [1.64, 2.70]	0.075
ECG parameters							
Heart rate, bpm	69 [62, 78]	68 [61.5, 78.5]	70 [62, 77.75]	0.696	68.5 [63, 77.75]	70 [62, 77]	0.774
PR interval, ms	166 [154, 188]	164 [152, 190]	166 [154, 187.5]	0.966	183 [165.5, 203]	175 [154, 195.5]	0.017
QRS, ms	86 [82, 96]	88 [81, 96]	86 [82, 96]	0.411	89 [82, 112]	86 [80.5, 97.5]	0.296
QTc, ms	450 [429.5, 469]	448 [430, 466.5]	451 [429, 469]	0.681	456 [443.75, 476.75]	452 [432, 471.25]	0.047
TTE parameters before ablation							
LAD, mm	39 [36, 43]	39 [37, 42]	39 [36, 43]	0.576	40 [37, 44]	38 [36, 42]	0.015
LALRD, mm	43 [40, 46]	42 [40, 46.5]	43 [40, 46]	0.738	45 [41.75, 47.25]	42 [39, 45]	<0.0001
LASID, mm	56 [52, 60]	56 [52, 60]	56 [52, 61]	0.892	58 [54.75, 64]	54 [51, 60]	<0.0001
LAV, mL	48.29 [40.51, 61.03]	48.69 [40.72, 60.56]	48.01 [40.04, 61.83]	0.831	54.21 [44.51,69.94]	45.36 [37.97, 58.12]	<0.0001
LAVI, mL/m^2^	26.7 [21.63, 33.24]	26.48 [21.64, 32.96]	26.96 [21.56, 33.52]	0.796	29.12 [24.8,36.1]	25.59 [20.71, 32.3]	<0.0001
LASI	0.76 [0.74, 0.79]	0.77[0.74, 0.80]	0.76 [0.73, 0.79]	0.226	0.77 [0.74,0.79]	0.76 [0.73, 0.79]	0.507
LVEF, %	58 [55, 59]	58 [57, 59]	58 [55, 59]	0.861	56.5 [52, 58.25]	58 [58, 59]	<0.0001
LVEDD, mm	47[45,50]	48[45,50]	47 [44,50]	0.462	48 [46,51]	47 [44, 50]	0.009
TTE parameters after ablation							
LAD, mm	38 [36, 41]	38 [36, 41]	38 [35, 41]	0.179	36 [33, 39]	39 [36, 42]	<0.0001
LALRD, mm	41 [38, 44.5]	41 [39, 44]	41 [38, 45]	0.374	39 [36, 43]	42 [39,45]	<0.0001
LASID, mm	53 [50, 57]	54 [50, 57]	53 [50, 57]	0.276	51 [47, 55]	54 [51,58]	<0.0001
LAV, mL	44.05 [35.52, 53.9]	45.82 [37.02, 54.72]	43.54 [34.87, 52.79]	0.149	37.03 [29.92, 46.4]	45.74 [38.8, 55.42]	<0.0001
LAVI, mL/m^2^	24.22 [19.56, 28.57]	24.22 [19.82, 29.37]	24.24 [19.39, 28.31]	0.405	20.12 [16.82, 25.76]	25.19 [20.86, 30.03]	<0.0001
LASI	0.78 [0.75, 0.8]	0.77 [0.74, 0.8]	0.78 [0.75, 0.80]	0.27	0.78 [0.74, 0.8]	0.78 [0.75, 0.8]	0.37
LVEF, %	59 [58, 60]	59 [58, 60]	59 [58, 60]	0.5	59 [58, 60]	59 [58, 60]	0.795
LVEDD, mm	47 [44, 50]	47 [45, 50]	47 [44, 50]	0.255	47 [44, 50]	47 [44, 50]	0.982
LARR, n(%)	94 (28.06)	26 (24.76)	68 (29.57)	0.364	–	–	–
Time interval, months	29.53 [15.23, 42.53]	22.23 [13.55, 40.60]	34.22 [17.17, 43.15]	0.015	23.97 [12.03, 42.18]	32.8 [17.4, 43.07]	0.026
AF recurrence, n(%)	105 (31.34)	–	–	–	26 (27.66)	79 (32.78)	0.364
Medications							
AADs, n(%)^†^	244 (72.84)	83 (79.05)	161 (70.0)	0.084	75 (70.79)	169 (70.12)	0.074
β-blockers, n(%)	68 (20.3)	15 (14.29)	53 (23.04)	0.065	19 (20.21)	49 (20.33)	0.981
RAAS blockers, n(%)^‡^	120 (35.82)	43 (40.95)	77 (33.48)	0.186	31 (32.98)	89 (36.93)	0.498
BNP concentrations							
Pre-ablation, pg/mL	108.04 [45.85,208.5]	70.19 [42.58, 239.97]	136.65 [49.53, 249.98]	0.094	115.31 [48.51, 217.06]	102.62 [45.26, 238.16]	0.85
Post-ablation, pg/mL	65.63 [34.0, 142.47]	56.24 [24.89, 159.56]	73.31 [48.27, 131.27]	0.203	51.76 [35.0, 89.66]	74.18 [37.56, 139.8]	0.087
Corin concentrations							
Pre-ablation, pg/mL	401.49 [225.42, 572.61]	511.63 [334.1, 672.56]	380.63 [191.7, 526.73]	<0.0001	442.85 [241.25, 631.68]	393.82 [207.09, 558.11]	0.036
Post-ablation, pg/mL	484.46 [335.67, 674.98]	538.28 [327.34, 628.95]	466.92 [327.34, 628.95]	0.004	516.83 [362.4, 697.84]	473.77 [320.88, 665.75]	0.047

LARR, left atrial reverse remodeling; AF, atrial fibrillation; CA, catheter ablation; BMI, body mass index; BSA, body surface area; PaAF, paroxysmal atrial fibrillation; LVH, left ventricular hypertrophy; T2DM, type 2 diabetes mellitus; HF, heart failure; CAD, coronary artery disease; eGFR, estimated glomerular filtration rate; TC, total cholesterol; TG, triglyceride; LDL-C, low-density lipoprotein cholesterol; HDL-C, high-density lipoprotein cholesterol; ECG, electrocardiogram; QTc, corrected QT interval; TTE, transthoracic echocardiography; LAD, left atrial diameter; LALRD, left-right diameter of left atrium; LASID, superior-inferior diameter of left atrium; LAV, left atrial volume; LAVI, left atrial volume index; LASI, left atrial sphericity index; LVEF, left ventricular ejection fraction; LVEDD, left ventricular end-diastolic diameter; AADs, antiarrhythmic drugs; RAAS, renin aniotension aldosterone system; BNP, type B natriuretic peptide.

^†^Including amiodarone, propafenone and dronedarone.

^‡^Including angiotensin-converting enzyme inhibitor, angiotensin receptor blocker and angiotensin receptor/neprilysin inhibitor.

### Pre-/post-procedural plasma corin levels in LARR and non-LARR patients treated with CA for AF

**Figure 2:**
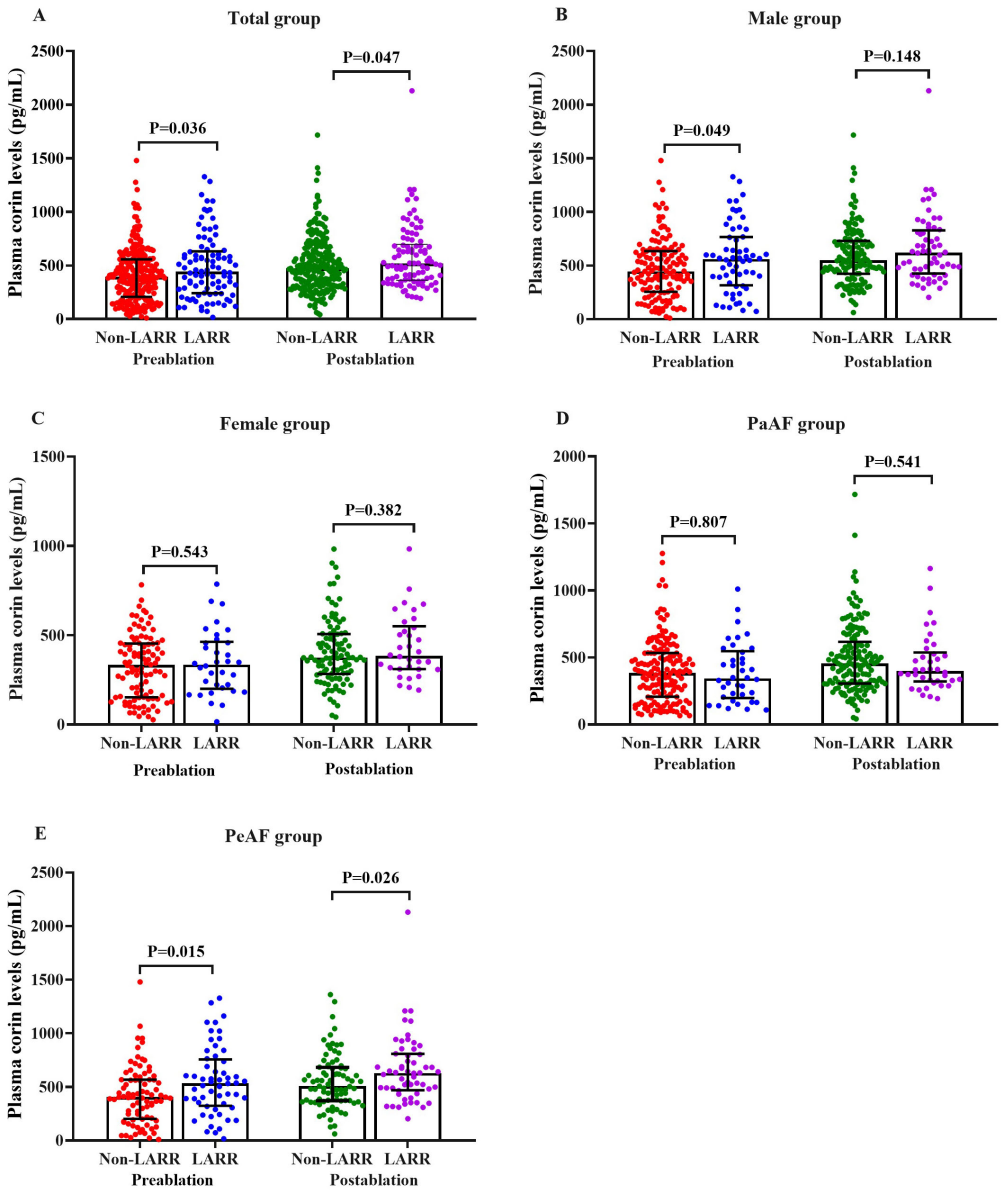
Pre- and post-procedural plasma corin levels in LARR and non-LARR patients treated with catheter ablation for atrial fibrillation. **(A)** Total group, **(B)** male subgroup, **(C)** female subgroup, **(D)** PaAF subgroup, and **(E)** PeAF subgroup. LARR, left atrial reverse remodeling; PaAF, paroxysmal atrial fibrillation; PeAF, persistent atrial fibrillation. The bar represents the 25%–75% range of values.

In the total cohort, the plasma corin concentrations drawn pre- and post-procedure in the LARR group were 442.85 (241.25, 631.68) and 516.83 (362.4, 697.84) pg/mL, respectively, and those were significantly higher than those in the non-LARR group [393.82 (207.09, 558.11) and 473.77 (320.88, 665.75) pg/mL; *P* = 0.036 and *P* = 0.047, respectively] (**Table 2**, **Figure 2A**).

**Table 2:** Pre/postprocedural plasma corin levels in LARR and non-LARR patients treated with catheter for atrial fibrillation.

Plasma corin levels		Non-LARR group	LARR group	*P* value
Total group				
	n=335	n=241	n=94	
Pre-ablation, pg/mL	401.49 [225.42, 572.61]	393.82 [207.09, 558.11]	442.85 [241.25, 631.68]	0.036
Post-ablation, pg/mL	484.46 [335.67, 674.98]	473.77 [320.88, 665.75]	516.83 [362.4, 697.84]	0.047
Sex subgroup				
Male	n=199	n=139	n=60	
Pre-ablation, pg/mL	474.74 [276.58, 647.15]	445 [254.5, 634]	559.3 [315.9, 765.7]	0.049
Post-ablation, pg/mL	554.62 [422.87, 748.86]	548.9 [422.9, 730.4]	620.5 [426.1, 828.2]	0.148
Female	n=136	n=102	n=34	
Pre-ablation, pg/mL	333.65 [169.38, 456.44]	333.7 [152.9, 453.4]	335.7 [200.7, 463.5]	0.543
Post-ablation, pg/mL	374.93 [288.31, 508.44]	374.6 [283.5, 506.8]	384.9 [310.5, 550.9]	0.382
AF type subgroup				
PaAF	n=195	n=157	n=38	
Pre-ablation, pg/mL	381.69 [205.85, 533.42]	383.4 [207.1, 532.8]	342.6 [197.9, 546.4]	0.807
Post-ablation, pg/mL	441.83 [306.68, 596.39]	455.2 [305.4, 616.9]	398.5 [321.2, 536.9]	0.541
PeAF	n=140	n=84	n=56	
Pre-ablation, pg/mL	531.57 [246.69, 621.13]	404.6 [202.0, 566.9]	533.6 [324.0, 757.0]	0.015
Post-ablation, pg/mL	552.77 [379.99, 719.63]	505.9 [370.7, 682.3]	629.3 [469.1, 807.9]	0.026

LARR, left atrial reverse remodeling; AF, atrial fibrillation; PaAF, paroxysmal atrial fibrillation; PeAF, persistent atrial fibrillation.

In a subgroup analysis of male patients, the plasma corin concentrations before CA were significantly higher in patients with LARR than in those without LARR [559.3 (315.9, 765.7) *vs*. 445 (254.5, 634) pg/mL; *P* = 0.049], but this was not found in post-ablation corin concentrations [620.5 (426.1, 828.2) *vs*. 548.9 (422.9, 730.4) pg/mL; *P* = 0.148; **Table 2**, **Figure 2B**]. However, in female patients, there was no difference between the LARR group and the non-LARR group both in pre- and post-ablation corin concentrations [pre-ablation: 335.7 (200.7, 463.5) *vs*. 333.7 (152.9, 453.4) pg/mL, *P* = 0.543; post-ablation: 384.9 (310.5, 550.9) *vs*. 374.6 (283.5, 506.8) pg/mL, *P* = 0.382; **Table 2**, **Figure 2C**].

In the PeAF subgroup, the corin concentrations were significantly higher in patients with LARR than in those without LARR [pre-ablation: 533.6 (324.0, 757.0) *vs*. 404.6 (202.0, 566.9) pg/mL, *P* = 0.015; post-ablation: 629.3 (469.1, 807.9) *vs*. 505.9 (370.7, 682.3) pg/mL, *P* = 0.026; **Table 2**, **Figure 2E**]. However, the corin levels between the LARR group and the non-LARR group was not found in PaAF patients [pre-ablation: 342.6 (197.9, 546.4) *vs*. 383.4 (207.1, 532.8) pg/mL; *P* = 0.807; post-ablation: 398.5 (321.2, 536.9) *vs*. 455.2 (305.4, 616.9) pg/mL, *P* = 0.541; **Table 2**, **Figure 2D**].

### Plasma corin threshold of best predictive value for LARR in AF patients treated with CA

**Figure 3:**
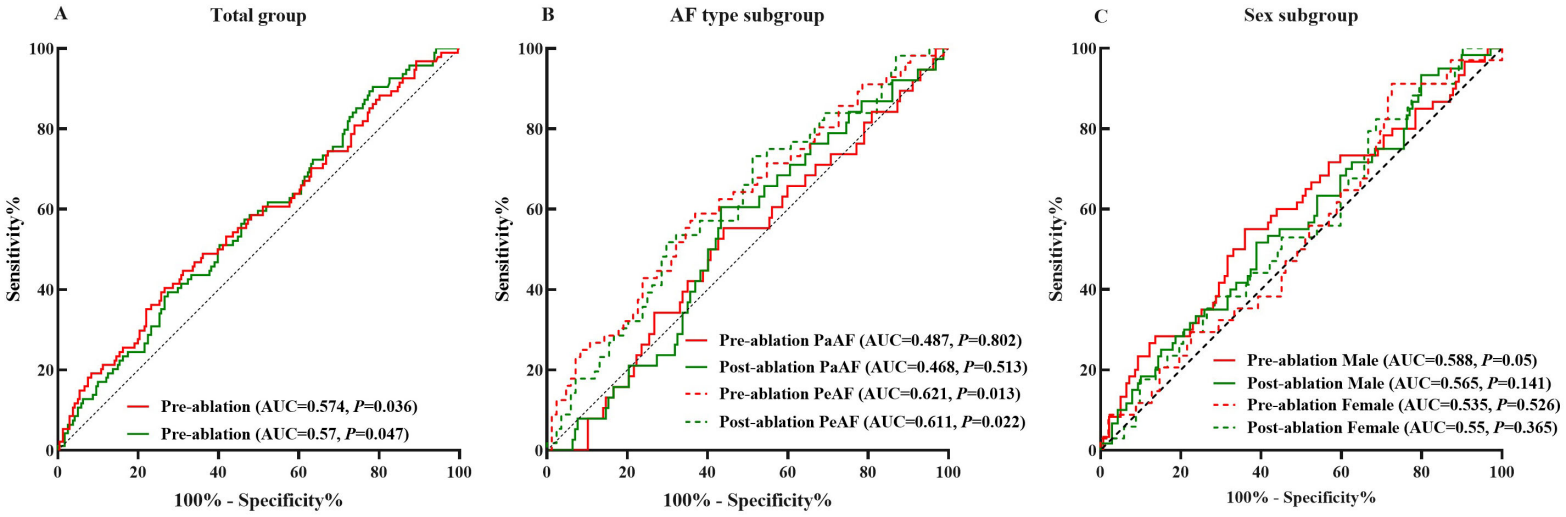
Plasma corin threshold of best predictive value for LARR in atrial fibrillation treated with catheter ablation. **(A)** Total group, **(B)** subgroup analysis of atrial fibrillation type, and **(C)** subgroup analysis of sex. LARR, left atrial reverse remodeling; PaAF, paroxysmal atrial fibrillation; PeAF, persistent atrial fibrillation; AUC, area under the curve.

The pre-ablation ROC curve analysis of plasma corin drawn was >535.22 pg/mL, and that of post-ablation was >307.69 pg/mL. These represented the best predictive values for LARR in AF patients after CA. The pre-ablation sensitivity was 40.4%, specificity was 73.4%, and area under the curve (AUC) was 0.574. At post-ablation, sensitivity was 90.4%, specificity was 21.6%, and AUC was 0.570 (*P* = 0.036, *P* = 0.047, respectively) (**Table 3**, **Figure 3A**).

**Table 3:** Plasma corin threshold of best predictive value for LARR in atrial fibrillation patients treated with catheter ablation.

	Total		PaAF		PeAF		Male		Female	
	Pre-ablation	Post-ablation	Pre-ablation	Post-ablation	Pre-ablation	Post-ablation	Pre-ablation	Post-ablation	Pre-ablation	Post-ablation
Area under curve (95%CI)	0.574 (0.504-0.643)	0.570 (0.502-0.637)	0.487 (0.386-0.588)	0.468 (0.371-0.564)	0.621 (0.526-0.716)	0.611 (0.516-0.706)	0.588 (0.5-0.676)	0.565 (0.479-0.651)	0.535 (0.426-0.644)	0.55 (0.441-0.66)
Threshold (pg/mL)	535.22	307.69	106.48	324.56	457.84	625.24	538.83	328.52	163.9	306.93
Sensitivity (%)	40.4	90.4	100	76.3	58.9	51.8	55.0	93.3	91.2	82.4
Specificity (%)	73.4	21.6	10.19	31.8	63.1	70.2	64.0	20.1	27.5	31.4
*P* value	0.036	0.047	0.802	0.513	0.013	0.022	0.05	0.141	0.526	0.365

PaAF, paroxysmal atrial fibrillation; PeAF, persistent atrial fibrillation.

In PeAF patients, the pre-ablation threshold was >457.84 pg/mL and the post-ablation threshold was >625.24 pg/mL. The pre-ablation sensitivity was 58.9%, and specificity was 63.1% (*P* = 0.013). In contrast, the post-ablation sensitivity was 51.8%, and specificity was 70.2% (*P* = 0.022). The pre-ablation AUC was 0.621, and the post-ablation AUC was 0.611 (**Table 3**, **Figure 3B**). However, the predictive value of corin levels in PaAF, male, or female subgroups, respectively, before and after ablation was not identified (**Table 3**, **Figures 3B, C**).

### Relationship between plasma corin levels and LARR in AF patients treated with CA

**Figure 4:**
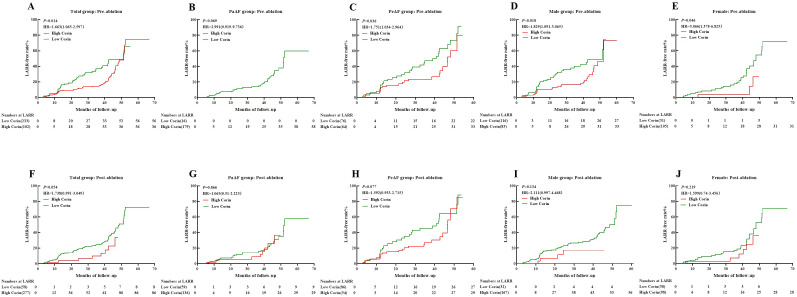
Kaplan–Meier curves showing LARR-free in atrial fibrillation treated with catheter ablation for different corin levels. **(A–E)** Corin levels pre-ablation in total, PaAF, PeAF, male, and female groups; **(F–J)** Corin levels post-ablation in total, PaAF, PeAF, male, and female. LARR, left atrial reverse remodeling; PaAF, paroxysmal atrial fibrillation; PeAF, persistent atrial fibrillation; HR, hazard ratio.

We classified low and high corin levels according to thresholds in different groups. The Kaplan–Meier survival curve showed that high corin concentrations measured before the procedure indicated a significantly higher LARR than low corin concentrations in the total group, PeAF group, male group, and female group (*P* = 0.014, 0.036, 0.018, and 0.046, respectively, **Figures 4A, C–E**), other than the PaAF group (*P* = 0.069, **Figure 4B**). However, post-ablation corin concentrations were not associated with LARR, whether in total group, different types of AF, or different sex (**Figures 4B–J**).

Cox regression and forest plot found that the plasma corin levels before or after the procedure, whether in total group, PaAF, PeAF, male, or female, were not associated with LARR in AF patients after CA with or without adjusting for confounding factors (**Supplementary Table S1**, **Supplementary Figure S1**).

### Independent predictors of LARR in AF patients treated with CA

**Figure 5:**
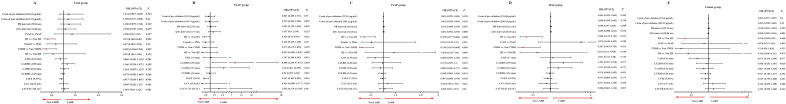
**(A–E)** Predictors of left atrial reverse remodeling in atrial fibrillation patients after catheter ablation in adjusted models. The continuous variables were normalized by Z-score, and one standard deviation was used for odds ratio calculation. OR, odds ratio; CI, confidence interval; LARR, left atrial reverse remodeling; AF, atrial fibrillation; PaAF, paroxysmal atrial fibrillation; PeAF, persistent atrial fibrillation; QTc, corrected QT; HT, hypertension; T2DM, type 2 diabetes mellitus; HF, heart failure; LAD, left atrial diameter; LALRD, left-right diameter of left atrium; LASID, superior–inferior diameter of left atrium; LAV, left atrial volume; LAVI, left atrial volume index; LVEF, left ventricular ejection fraction; LVEDD, left ventricular end-diastolic diameter. All ultrasound indicators are from baseline.

In adjusted models, plasma corin levels before or after CA, whether in the total group, PaAF, PeAF, male, or female, did not predict LARR in AF patients after CA (**Figure 5**). In the total group, absence of hypertension (*P* = 0.001, OR = 0.33, 95% CI: 0.168–0.649) and T2DM (*P* = 0.007, OR = 0.269, 95% CI: 0.104–0.701) was associated with a greater likelihood of LARR in AF patients after CA (**Figure 5A**). The same results were found in the PeAF group (absence of hypertension: *P* = 0.008, OR = 0.229, 95% CI: 0.077–0.684, and T2DM: *P* = 0.009, OR = 0.136, 95% CI: 0.031–0.606; **Figure 5C**) and the male group (absence of hypertension: *P* = 0.011, OR = 0.306, 95% CI: 0.122–0.764, and T2DM: *P* = 0011, OR = 0.207, 95% CI: 0.061–0.702; **Figure 5D**). In the female group, only absence of hypertension was associated with a greater likelihood of LARR in AF patients after CA (*P* = 0.048, OR = 0.314, 95% CI: 0.099–0.99; **Figure 5E**). In the PaAF group, absence of hypertension (*P* = 0.041, OR = 0.353, 95% CI: 0.13–0.957), higher LALRD (*P* = 0.029, OR = 8.408, 95% CI: 1.239–57.071), and lower LVEF (*P* = 0.016, OR = 0.367, 95% CI: 0.161–0.832) baseline were associated with a greater likelihood of LARR in AF patients after CA (**Figure 5B**).

## Discussion

This is the first study to investigate the association between plasma corin levels before and after CA for AF and LARR. Our primary findings are as follows: (1) Plasma corin concentrations pre- or post-ablation were significantly higher in patients with LARR than in those without LARR in general population; (2) the Kaplan–Meier survival curve showed that high corin concentrations measured before CA indicated a significantly higher rate of LARR than low corin concentrations in the total, PeAF, male, and female groups, except for the PaAF group; (3) whether or not adjusted for confounding factors, the predictive value of plasma corin levels in LARR before or after ablation was not evident; and (4) absence of hypertension and T2DM was associated with a greater likelihood of LARR in AF patients after CA.

### LARR and AF ablation

LA structural remodeling, characterized by atrial dilation and progressive fibrosis, is a key pathological feature in the development and maintenance of AF. The concept of LARR has emerged as a therapeutic goal aimed at restoring atrial structure and function (29). Various studies have assessed the effects of AF ablation on LA structure and function. Most studies noted a significant reduction in LAV only in the SR group, with a significant improvement in LA active and reservoir functions but not in the AF recurrence group (8, 30–34). Our study also confirmed LAVI reduction in the entire sample after the first CA of AF. However, a difference in LAVI reduction was observed regardless of AF recurrence. Several studies have also found no association between success in ablation and atrial reduction (35–37), which is consistent with our findings. This may be explained by AF burden reduction using CA despite AF recurrence (36, 37), undetected AF recurrent patients categorized into the success group (36), the baseline LA size, AF type, and the extent of scarring following CA (38).

### Plasma corin levels and LARR in AF patients treated with CA

The atrial remodeling process is associated with levels of ANP and BNP (39, 40), reflecting neurohormonal activation in response to atrial stress. Natriuretic peptides increase in the event of AF due to atrial stretch. However, longstanding AF leads to atrial structural remodeling, resulting in reduced NP production capacity due to cardiomyocyte loss and fibrosis progress (10). Several studies have reported that LARR after CA likely occurred in AF patients with higher pre-ablation ANP/BNP levels (9, 10, 41). In addition, the preprocedural ANP/BNP ratio was a robust predictor of LARR after SR restoration using rhythm-control therapy in PeAF patients (9). Circulating ANP/N-terminal proANP/mid-regional proANP levels are valuable in predicting AF development and recurrence as well as LARR after CA (10, 42–45). Corin has been identified as the only physiological proANP convertase (46). ProANP is co-localized with corin in cardiomyocytes (47, 48), and for it, cleavage by corin is dominant (29). Therefore, the role of corin in atrial diseases such as atrial arrhythmias, atrial enlargement, or mitral stenosis, may be more pivotal than in HF or coronary heart disease (18). Based on this speculation, one study by our group demonstrated that plasma corin concentrations were significantly higher in AF patients than in controls, and those in PeAF patients were much higher than those in PaAF patients (19). Therefore, the increasing trend of plasma corin levels in AF patients is consistent with that of ANP levels (19, 29). Furthermore, corin and ANP levels are also significantly elevated in patients with a higher AF burden compared with those with a lower AF burden (10).

Another prospective observational study involving 616 patients undergoing first CA of AF found that the pre-ablation corin concentration of the recurrence group was significantly higher than that of the non-recurrence group (20). Elevated pre-ablation corin levels were significantly associated with an increased risk of AF recurrence after CA (20). In our study, although plasma corin concentrations drawn pre- and post-ablation in the LARR group were higher than those in the non-LARR group, the difference was reduced after adjusting for confounding factors. This indicates that plasma corin levels provide minimal prognostic utility in LARR after CA of AF. There are several possible explanations. First, several studies enrolled AF patients with LA enlargement (LAVI ≥ 34 mL/m^2^) (9, 49, 50), whereas the baseline median LAVI in our study was 26.7 mL/m^2^. Our patients had less pronounced atrial remodeling compared to other studies. Therefore, we defined LARR as a ≥20% reduction in LAVI, differing from the 15% cutoff used in prior studies (9, 10, 49). Besides that, we did not find a significant difference in BNP concentrations of baseline before and after CA. This may occur because less atrial remodeling reduces atrial wall stretch, resulting in diminished shedding of corin from atrial cardiomyocytes and natriuretic peptide secretion. Second, numerous studies have demonstrated LARR due to restoration and maintenance of SR after CA (10, 38, 41, 50–53). Meta-analysis revealed that LAD, LAV, and LAVI were significantly decreased by ablation (54). The degree of LARR depends on the difference in LA compliance (55). LA is more compliant and stretched, with preserved LA myocytes and less fibrosis, resulting in greater reversibility from structural remodeling (10). However, we failed to demonstrate reduced AF recurrence rates among patients exhibiting LARR after CA. A plausible explanation is that AF burden decreased significantly after ablation despite AF recurrence (37). LARR may have shown a pronounced correlation with a reduction in AF burden rather than AF recurrence. Third, majority of the studies employed follow-up periods ranging from 6 to 12 months, whereas 29.53 months was used to assess extended-term LARR post-ablation in our study. Plasma corin levels may have limited predictive utility for long-term LARR following AF ablation. Atrial cardiomyopathy (ACM) is increasingly recognized as a key contributor to the development and perpetuation of AF and involves complex structural, electrical, and functional remodeling of the atrial myocardium (56, 57). Future studies concurrently consider ACM and other emerging biomarkers which can predict atrial remodeling and may help to enhance the predictive value of corin for LARR.

### Hypertension

We found that absence of hypertension was associated with a higher rate of LARR, which is consistent with most studies. One study showed that absence of hypertension was an independent predictor of significant LAV reduction following ablation (35). Another study found that younger patients without hypertension are most likely to experience a reduction in LAVI following cryoballoon-based pulmonary vein isolation (58). Furthermore, anti-hypertensive therapy has been shown to prevent LA dilatation and favorable reverse remodeling (59). Hypertension induces atrial remodeling and represents an independent risk factor for AF. Hypertension increases left ventricular afterload and end-diastolic pressure (60), leading to atrial enlargement and dysfunction, interstitial fibrosis, inflammation, heterogeneous conduction, and a greater propensity for AF (61, 62). Therefore, hypertension plays a central role in initiating and perpetuating adverse LA remodeling.

### Diabetes mellitus

We found that absence of T2DM was also associated with a higher rate of LARR. A study including 204 consecutive AF patients who underwent first CA found that patients with T2DM had significantly reduced LA function and increased LA stiffness compared with those without T2DM (63). T2DM was associated with reduced LA function in patients with hypertrophic cardiomyopathy (64), hypertension (65), and significant aortic regurgitation (66).

The underlying mechanisms by which T2DM contributes to atrial remodeling are not completely understood. Several hypotheses have been proposed. First, abnormal glucose metabolism activates enhanced angiotensin II and transforming growth factor-β signaling, fostering a pro-fibrotic environment that ultimately leads to LAR (67). Second, autonomic nervous system imbalance, characterized by sympathetic nervous system predominance in individuals with hyperglycemia, can contribute to LAR. Autonomic dysfunction, which is highly prevalent in diabetes patients, impacts atrial size via alterations in sympathetic and parasympathetic neural activity (67). Third, enhanced inflammation in abnormal glucose metabolism can be associated with LA remodeling (63). Furthermore, microvascular ischemia resulting from the metabolic derangements of diabetes represents an additional contributing factor (65).

Therefore, diabetes contributes to LAR through a complex interplay of metabolic disturbances, oxidative stress, inflammation, mitochondrial dysfunction, and neurohumoral activation. These mechanisms collectively promote structural and functional alterations in LA, including fibrosis, impaired compliance, and dysfunctional contractility (67).

### Study limitations

Several limitations of this study should be noted. First, as a single-center observational study, our findings may be influenced by residual confounding factors inherent in this design paradigm. External validation through multicenter trials with larger cohorts is warranted to confirm these observations. Second, information on post-procedural AF burden is not uniformly available, which can affect LARR. Third, using cardiac magnetic resonance imaging to evaluate LAV is more accurate than TTE, and it can also determine LA scarring. Fourth, the study focused exclusively on LAVI quantification without LA reservoir function and strain analysis, which may provide deeper insights into post-ablation structural changes. Fifth, the potential correlation between ANP levels and LARR was not explored. The combined evaluation of circulating ANP and corin levels may serve as a new panel for LARR post-ablation, warranting future investigation. Sixth, thermal procedures may lead to ischemia, coagulation necrosis, edema, and local inflammation of atrial tissue, which may result in the increased shedding of corin from atrial myocytes. However, we did not dynamically monitor the changes in corin level after ablation, for example, at the first, third, sixth, ninth, or 12th month after ablation. Serial measurements at later time points could potentially yield different and more meaningful insights into the relationship between corin and reverse remodeling. Seventh, it is important to note that the relatively low AUC values (0.57–0.62) indicate poor discriminatory power, and the use of dichotomized variables in survival analysis based on weak cutoffs has some limitations and can reduce statistical power. Eighth, AF duration was not be analyzed in this retrospective cohort. The lack may introduce residual confounding into our multivariate models. Future studies should include the parameter to validate and extend our findings.

## Conclusions

Although plasma corin concentrations drawn pre- and post-ablation in the LARR group were higher than those in the non-LARR group, the difference was reduced after adjusting for confounding factors. Plasma corin levels provide minimal prognostic utility in LARR after CA of AF. The absence of hypertension and T2DM is associated with a greater likelihood of LARR.

## Data Availability

The original contributions presented in the study are included in the article/**Supplementary Material**. Further inquiries can be directed to the corresponding authors.
