# Supplementary material for: Plasma corin levels provide minimal prognostic utility in left atrial reverse remodeling after catheter ablation of atrial fibrillation: an observational study
Source: Front Endocrinol (Lausanne). 2025 Nov 24;16:1717422. doi: 10.3389/fendo.2025.1717422 (PMC12682628; doi:10.3389/fendo.2025.1717422)

**Supplementary Table 1 Cox regression analysis for corin concentrations between LARR and non-LARR in atrial fibrillation patients treated with catheter ablation**

| Corin concentrations (Per SD, pg/mL) | Unadjusted | | | Adjusted^*^ | | |
| --- | --- | --- | --- | --- | --- | --- |
|  | HR | 95%CI | *P* | HR | 95%CI | *P* |
| Total group | | | | | | |
| Pre-ablation (264.32) | 1.203 | 0.998-1.45 | 0.053 | 1.001 | 1.0-1.002 | 0.236 |
| Post-ablation (267.27) | 1.167 | 0.963-1.415 | 0.114 | 1.0 | 0.998-1.001 | 0.657 |
| PaAF group | | | | | | |
| Pre-ablation (233.54) | 0.911 | 0.652-1.272 | 0.582 | 1.001 | 0.999-1.004 | 0.422 |
| Post-ablation (246.74) | 0.828 | 0.576-1.191 | 0.309 | 0.998 | 0.995-1.0 | 0.103 |
| PeAF group | | | | | | |
| Pre-ablation (297.01) | 1.25 | 0.983-1.589 | 0.069 | 1.0 | 0.999-1.002 | 0.86 |
| Post-ablation (282.1) | 1.219 | 0.957-1.553 | 0.11 | 1.0 | 0.999-1.002 | 0.549 |
| Male group |  |  |  |  |  |  |
| Pre-ablation (291.68) | 1.216 | 0.96-1.542 | 0.105 | 1.0 | 0.999-1.002 | 0.545 |
| Post-ablation (286.43) | 1.142 | 0.892-1.462 | 0.291 | 1.0 | 0.998-1.002 | 0.974 |
| Female group |  |  |  |  |  |  |
| Pre-ablation (176.6) | 1.031 | 0.74-1.436 | 0.858 | 1.002 | 0.999-1.006 | 0.176 |
| Post-ablation (181.35) | 1.096 | 0.796-1.509 | 0.573 | 1.001 | 0.997-1.004 | 0.703 |
| SD, standard deviation; HR, hazard ratio; CI, confidence interval; PaAF, paroxysmal atrial fibrillation; PeAF, persistent atrial fibrillation; ^*^Adjusted for AF type, hypertension, type 2 diabetes mellitus, heart failure, PR interval, corrected QT interval, and transthoracic echocardiography parameters before ablation (left atrial diameter, left-right diameter of left atrium, superior-inferior diameter of left atrium, left atrial volume, left atrial volume index, left ventricular ejection fraction, left ventricular end-diastolic diameter). | | | | | | |

**Supplementary figure legends**

**Supplementary Figure 1 Hazard ratios with 95% confidence intervals for one standard deviation increase in corin concentration of left atrial reverse remodeling in atrial fibrillation patients after catheter ablation.** A, crude model; B, adjusted model. HR, hazard ratio; SD, standard deviation; CI, confidence interval; PaAF, paroxysmal atrial fibrillation; PeAF, persistent atrial fibrillation.


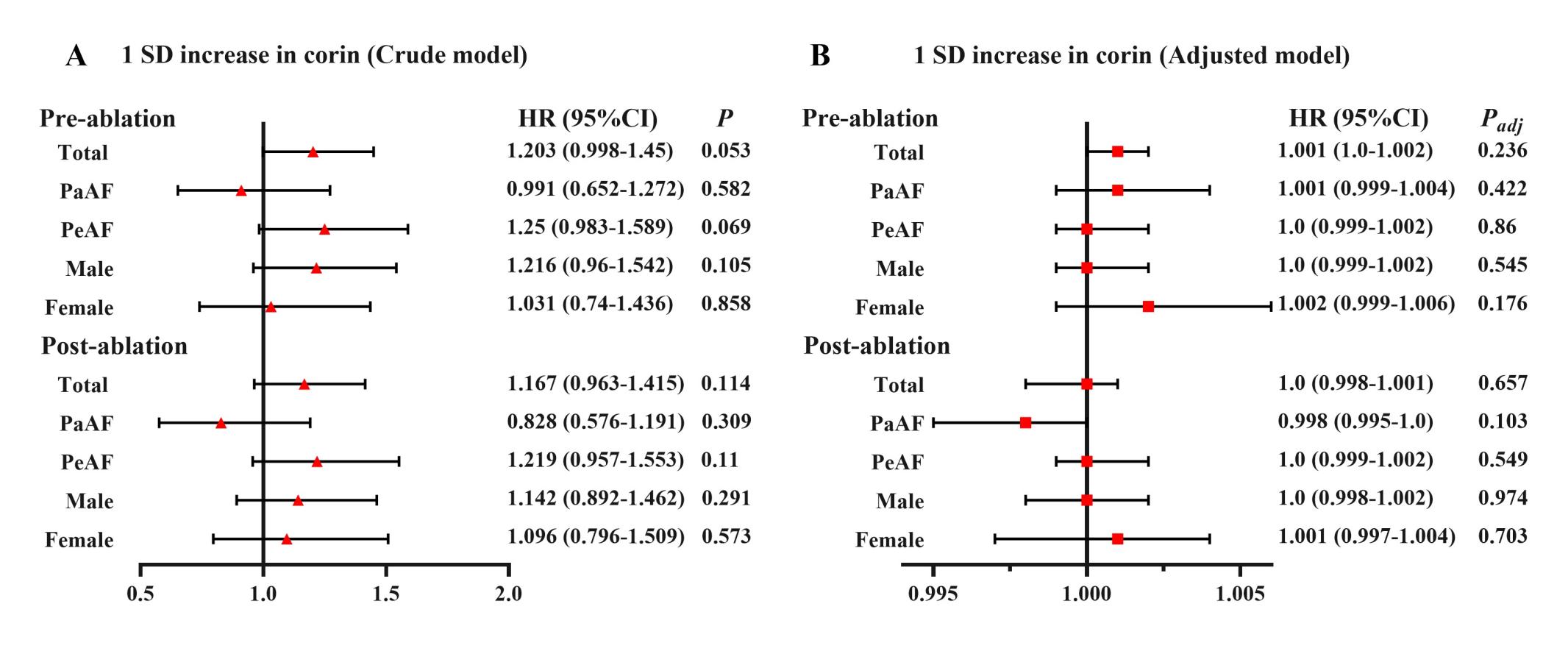

Supplement: Supplementary file 1 [file DataSheet1.docx]
